# Upregulation of Cytotoxic T-cells in pediatric patients with Gaucher disease

**DOI:** 10.1038/s41598-022-08843-4

**Published:** 2022-03-23

**Authors:** Asmaa M. Zahran, Khaled Saad, Khalid I. Elsayh, Madleen Adel A. Abdou, Amir M. Abo-Elgheet, Esraa M. Eloseily, Shaimaa M. Khalaf, Shabaan Sror, Faisal-Alkhateeb Ahmad, Amira Elhoufey, Aliaa Ghandour, Naglaa S. Osman

**Affiliations:** 1 Department of Clinical Pathology, South Egypt Cancer Institute, Assiut University, Assiut, Egypt; 2 Department of Pediatrics, Assiut University Hospital, Assiut, Egypt; 3 Department of Clinical Pathology, Assiut University Hospital, Assiut, Egypt; 4 Department of Community Health Nursing, Faculty of Nursing, Assiut University, Assiut, Egypt; 5 Department of Community Health Nursing, Alddrab University College, Jazan University, Jazan, Saudi Arabia; 6 Department of Medical Microbiology and Immunology, Faculty of Medicine, Assiut University, Assiut, Egypt

**Keywords:** Immunology, Medical research

## Abstract

Cytotoxic (CD8) T-cells and natural killer (NK) cells have a significant immune function role. The ongoing stimulation of immunity and the excessive release of proinflammatory cytokines observed in pediatric patients with Gaucher disease (GD) can affect immune cells. Few studies have looked at the proportion of cytotoxic CD8 T-cells and their subsets in children with GD. A prospective case–control study was performed involving twenty pediatric patients with type 1 GD and twenty healthy age-matched controls. All patients received regular enzyme replacement therapy (ERT) for at least 6 months before the study. Complete blood count and flow cytometric analyses of CD8 T, Tc1, Tc2, NK, and NK T-cells were performed. GD patients showed significantly increased of CD8 T, Tc1 and significantly decreased NK cells frequencies when compared to healthy controls. However, no significant difference in Tc2 and NK T-cells was found between the studied groups. GD patients on regular ERT have increased CD8+ T-cell frequencies, predominantly Tc1, together with a reduction in NK cells than in healthy controls. These crucial immunological changes may contribute to some extent to the pathogenesis and the progression of GD.

## Introduction

Gaucher disease (GD) is an inherited lysosomal storage disease with a defect in the GBA1 gene, resulting in an abnormal configuration and function of β-glucocerebrosidase^1^. The role of this enzyme is to break down glucosylceramide into glucose and ceramide; thus, the consequence of its defect is the buildup of glucosylceramide in the lysosomes of the monocyte/macrophage line of the reticuloendothelial system, resulting in the evolution of these cells into Gaucher cells, which boast a crumpled tissue paper appearance^1–3^. Three clinical forms of GD have been defined according to the existence or absence of any neurological manifestation^2,4^.

The complex pathophysiology of GD could be due to not merely the loading of monocyte and macrophage lysosomes with undegraded glucosylceramide; in addition, there are also remarkable immunological irregularities that might be the result of an interruption in the normal immunological role of lysosomes as well as the effect of accumulated glucosylceramide^1^. In GD, the previous research showed ongoing stimulation of the immune system with excessive release of proinflammatory cytokines as interleukin-1 (IL-1) and its receptor antagonists, IL-2, -6, -8, -10, and 18, as well as tumor necrosis factor, transforming growth factor, and macrophage colony-stimulating factor from the macrophage/monocyte lineage^5^. Besides, the alteration in the immune cell subsets is a critical element involved in the pathogenesis of all GD types^6^.

Two types of T-cells exist helper CD4 T-cells and cytotoxic CD8 T-cells. Cytotoxic CD8 cells have a significant role in the host's immune defense against intracellular pathogens, viruses, and tumors through the production of lytic substances^1^. They can be categorized based on their cytokine profile into multiple subsets, such as T cytotoxic 1 (Tc1), T cytotoxic 2 (Tc2), T cytotoxic 9 (Tc9), T cytotoxic 17 (Tc17), and CD8 T regulatory cells^7^. Among these, Tc1 cells are cytotoxic cells that secrete granzyme and perforin, which help in killing antigen-bearing cells. They also produce cytokines like interferon (INF)-γ and tumor necrosis factor–α, which hasten the effects of the immune system—namely, the innate and the adaptive responses against pathogens intracellularly^8^. Tc2 cells are less cytotoxic cells that have a major role in allergic and autoimmune diseases. Their cytokine profile is similar to some extent to that of the helper 2T-cells in that they produce IL-4, IL-5, and IL-13^7^. Disruption of the T-cell network has been observed with a decrease in both subtypes of T-cells in GD patients^9^.

Natural killer (NK) cells are considered one of the types of cytotoxic cells that have a vital part in the innate immune system's response. These cells identify lipid and/or glycolipid antigens when presented in association with MHCs like CD1d^10^. The ongoing stimulation of immunity and the excessive release of the proinflammatory cytokines detected in patients with GD are supposed to alter the function of the NK cells and reduce their count, which may predispose GD patients to cancer and infections^11,12^.

NK T-cells are a special subtype of T regulatory cells, named for their coexisting T-cell receptors and NK-cell markers on their surface^13^. Upon stimulation by signals, NK T-cells produce cytokines and participate in dendritic cells' activation as one of the antigen-presenting cells, enhancing their immune response^14^. Also, they regulate the immune response in autoimmune and inflammatory disorders and enhance the host's defense against infections and malignancy^15,16^.

Enzyme replacement therapy (ERT) is the mainline of management in GD, existing in three forms: imiglucerase (Cerezyme; Sanofi Genzyme, Cambridge, MA), velaglucerase alfa (Vpriv; Takeda Pharmaceuticals, Tokyo, Japan), and taliglucerase alfa (Elelyso; Pfizer, New York, NY)^17^. Regular intravenous infusions of ERT could result in significant clinical and hematological improvement^18^. It might also alter the immune response in GD patients, supposedly inducing humoral and cellular immunity changes that could lead to a loss of immune tolerance^19^. However, complete degrees of clinical and hematological improvement are still not achieved in many patients, which might explain the residual alteration in immune cells, clinical disease, and later complications^20,21^.

Few studies to date have looked at alterations in CD8 T-cell subsets depending on their cytokine profiles in GD pediatric patients. This study mainly focused on CD8+ T-cells, including two subsets (Tc1 and Tc2); NK cells; and NK T-cells in pediatric patients with type 1 GD receiving regular ERT.

## Patients and methods

This prospective case–control single-center study was conducted in the hematology department of Assiut Children's Hospital in Assuit, Asyut, Egypt. Our study was approved by the research ethics committee of Assiut University (No. 17300210). All methods and protocols of our work were performed in accordance with the relevant guidelines and regulations of Declaration of Helsinki and Assiut University. All caregivers of all participants have given their informed written consent.

Twenty GD patients were recruited for this study. All included GD patients were diagnosed by the recognition of decreased β-glucocerebrosidase enzyme activity in white blood cells by standard technique. All patients were diagnosed clinically and genetically with type I disease. All patients were on regular ERT in the form of an intravenous infusion of 60 IU of imiglucerase (Cerezyme) performed every two weeks for at least 6 months before the study. Patients with recent infection, any chronic immunosuppressive state, or any immunosuppressive drugs were excluded from this study. Twenty healthy matched controls were also included in this investigation for comparison purposes. Comprehensive history-taking, clinical assessments, and laboratory investigations were completed for all patients and controls. Venous blood samples were withdrawn from all patients prior to their regular Enzyme replacement therapy and used for complete blood count and for flow cytometric study to estimate the frequency of lymphocyte subsets, including CD8 T-, Tc1, Tc2, NK, and NK T-cells.

**Figure 1:**
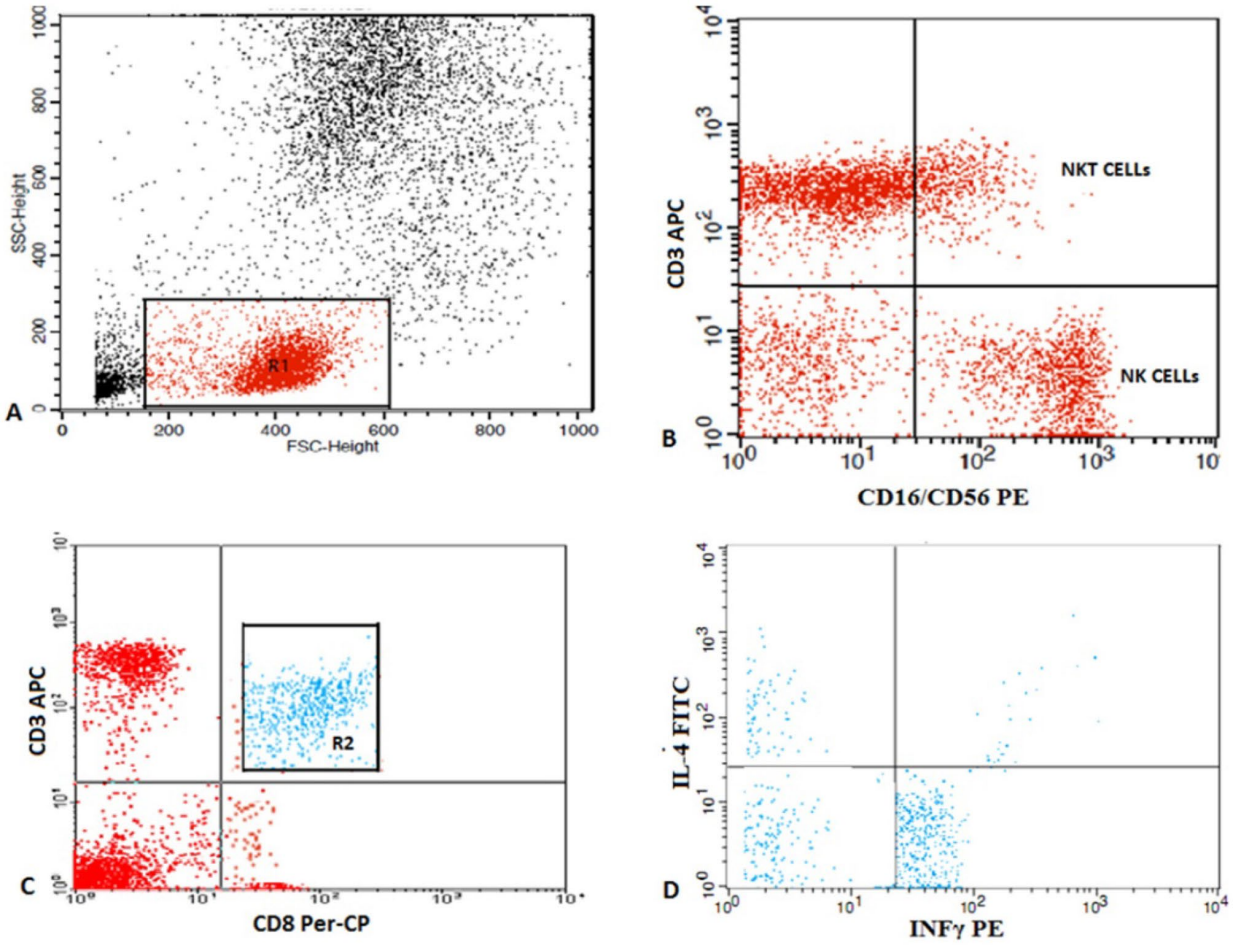
Flow cytometric detection of NK, NKT cells, and subtypes of T cytotoxic cells. (**A**) Scatter histogram was used to define the lymphocytes population (R1). (**B**) The expression of CD3 and CD16/CD56 on lymphocytes was assessed to detect NK and NKT cells. (**C**) The expression of CD8 was assessed on lymphocytes and then gated for further assessment of intracellular cytokines. (**D**) The expression of IL-4 and IFNγ on CD8 T cells. Tc1 cells (IFN-γ (+) IL-4(−) CD8 T cells), and Tc2 cells (IFN-γ (−) IL-4(+) CD8 T cells).

Fifty µL of blood was added to five µL of allophycocyanin (APC)-conjugated cluster of differentiation (CD)3 and phycoerythrin (PE)-conjugated CD16/56 (Becton Dickinson Biosciences, San Jose, CA). Following incubation for 15 min at 4 °C in the dark, red blood cell lysis, washing, and resuspension in phosphate-buffered saline (PBS) were completed. Flow cytometric study by FACSCaliber flow cytometry using the CellQuest software (Becton Dickinson Biosciences) was done. An anti-human immunoglobulin G serving as the isotype-matched negative control was used with every sample. To illustrate the different lymphocyte subsets, we used a scatter histogram. CD3+ (pan–T-cell marker), CD3 + CD16 + CD56 + (NK T-cell marker), and CD3 − CD16 + CD56 + (NK cell marker) were identified. For the detection of T cytotoxic cells, a blood sample of 300 µL was cultured in 300 µL of Roswell Park Memorial Institute 1640 medium (1:1) and incubated with 3 µL of phorbol myristate acetate (United States Biological, Salem, MA) and 1µL of ionomycin (United States Biological) as a positive polyclonal nonspecific stimulus for 12 h at 37 °C in a 5% CO_2_ incubator. The addition of 3 µL of brefeldin A (United States Biological) was performed instantaneously to prevent cytokine release at Golgi, permitting the ideal identification of molecules^12^. Then, 50μL of the prepared mixture was stained with peridinium–chlorophyll–protein (Per-CP)-conjugated CD8 and APC-conjugated CD3 (Becton Dickinson Biosciences) for 15 min at 4 °C. After red blood cell lysis and washing, a fixing solution was added, and incubation for 10 min was completed. Then, after washing with PBS, permeabilization solution was added with 5 µL of fluorescein isothiocyanate-conjugated IL-4 and PE-conjugated INF-γ (Becton Dickinson Biosciences) and left for 30 min at 4 °C. Finally, the cells were washed once and resuspended in PBS. The interpretation of the cells was performed by FACSCaliber flow cytometry using the CellQuest software. Twenty thousand measures were obtained. The scatter histogram (forward and side histograms) was drawn to illustrate different lymphocyte subsets. Then, proportions of cytotoxic CD8 T-cells were measured. Finally, the appearance of IL-4 and INF-γ on CD8 T-cells were identified. Tc1 cells [IFN-γ (+) IL-4 (−) CD8 T-cells], and Tc2 cells [IFN-γ (−) IL-4 (+) CD8 T-cells] were reported as proportions of the total CD8 T-cells (Fig. 1).

### Statistical analysis

The Statistical Package for the Social Sciences version 16.0 (IBM Corporation, Armonk, NY) was used to analyze the data. For continuous data, mean ± standard deviation values were calculated, while percentages were estimated for categorical data. An independent-samples t-test was used to analyze the variations between patients and controls. Spearman and Pearson's correlations established correlations between different variables. A p-value of less than 0.05 was considered to be statistically significant.

### Ethics approval

All protocols and investigations of our study followed the regulations of the research ethics committee of Assiut University (No. 17300210).

### Consent to participate

All caregivers of all participants have given their informed written consent.

## Results

The descriptive data of GD patients and controls are listed in Table 1. Regarding the genotyping of our patients, 65% have homozygous L444P mutation, and 35% have no data. Nine of the studied GD patients were boys (45%), and 11 were girls (55%). The body weight was reduced among GD patients relative to that in the healthy control group (p = 0.046). All patients had hepatosplenomegaly, and no splenectomy had been performed in any of the studied patients. The most frequent skeletal manifestations were osteopenia and Erlenmeyer flask deformity (Table 1).

**Table 1:** Descriptive clinical and laboratory data of GD patients.

Item	Patients n = 20	Control n = 20	*p*-value
Weight (Kg)	32.25 ± 12.6	42.1 ± 17.3	0.046*
Age (years)	10.8 ± 3.81	11.02 ± 3.87	0.086
Male/ Female	9/11	11/9	0.56
Family history (yes/no)	14/6	–	–
Liver span (cm)	11.47 ± 2.91	9.5 ± 1.68	0.02*
Spleen span (cm)	13.3 ± 5.04	7.5 ± 2.33	< 0.01*
**Skeletal involvement**			
Osteopenia	18/20		
Erlenmeyer flask deformity	3/20		
Fracture	1/20	–	–
Kyphoscoliosis	1/20		
Pigeon chest	1/20		
Recurrent chest infection	5/20	–	–
Interstitial pulmonary diseases	3/20	–	–
**Neurological manifestations**	14/20		
Squint	3/20	–	–
EEG changes	8/20		
Convulsion	6/20		
ß-Glucocerebrosidase (Mmol/gm/h) (before ERT)	0.32 ± 0.172	–	–
Chitotriosidase (Mmol/l/h) (before ERT)	3978.34 ± 4051.4	–	–

Values represent mean ± SD. Independent T-test is used in comparison between two groups.

*n* number, *Kg* kilogram, *cm* centimetre, *g* gram, *dL* decilitre, *L* liter, *WBCs* white blood cells, *Mmol* millimole, *h* hour, *ERT* enzyme replacement therapy.

**Figure 2:**
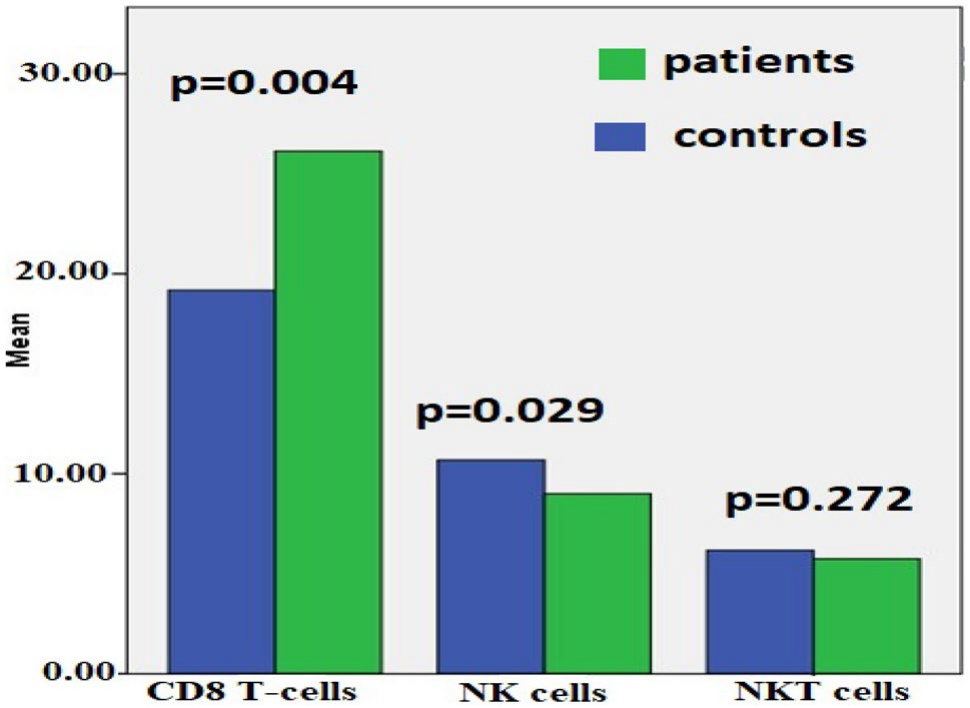
Percentages of CD 8, NK and NKT cells in patients versus control.

The studied lymphocyte subsets in GD patients and controls are listed in Table 2. A statistically significant increase in cytotoxic CD8 T-cells was observed in GD patients (p = 0.004, Fig. 2). Additionally, Tc1 cell counts were significantly elevated in GD patients in relation to controls (p = 0.025). Furthermore, a reduction in NK cells in GD patients was observed in comparison with controls (p = 0.029). However, no significant difference was noted concerning the numbers of NK T-cells between the studied groups. A significant positive correlation was observed between the chitotriosidase enzyme level and the CD8 cell count in GD patients, while a significant positive correlation existed between the liver and spleen span in GD patients and the counts of both CD8 cells and NK T-cells (Table 3).

**Table 2:** Peripheral blood, CD8 T cells, CD8 subtypes (Tc1, Tc2), NK and NKT cells in GD patients and control.

	Patients (n = 20)	Controls (n = 20)	*P* value
Hemoglobin (g/ dL)	10.91 ± .3.01	11.32 ± 1.15	0.06
Platelets (10^9^ cell/ L)	196.39 ± 73.1	222.10 ± 63.72	0.207
WBCs (10^9^ cell/ L)	6.67 ± 1.41	7.23 ± 2.01	0.343
CD8 T cells, (%)	26.133 ± 9.15	19.184 ± 3.105	0.004*
NK cells, (%)	9.001 ± 2.203	10.683 ± 2.462	0.029*
NKT cells, (%)	5.749 ± 1.621	6.176 ± 0.574	0.272
Tc1 cells, (%)	27.257 ± 8.475	21.719 ± 6.116	0.024*
Tc2 cells, (%)	2.614 ± 2.083	2.190 ± 0.960	0.416

Values represent mean ± SD of the relative count of each population. Independent T-test is used in comparison between two groups.

*n* number, *CD* cluster of differentiation, *(%)* percentage, *NK* natural killer, *NKT* natural killer T, *Tc1* T cytotoxic 1, *Tc2* T cytotoxic 2.

**Table 3:** Correlation between CD8 and NKT cells and different variables in GD patients.

Item	CD8		NKT	
	r- value	*p*-value	r- value	*p*-value
Chitotriosidase	0.476	0.038*	–	–
Liver span	0.703	0.001*	0.479	0.038*
Spleen span	0.510	0.026*	0.513	0.025*
NK	0.569	0.009*	0.599	0.005*
NKT	0.704	0.001*	–	–

Pearson’s correlation coefficient test is used to determine correlation.

*CD* cluster of differentiation, *NK* natural killer, *NKT* natural killer T.

## Discussion

The accumulation of undegraded glucosylceramide in patients with GD, resulting in splenomegaly with hypersplenism and bone marrow infiltration, may explain the disturbance of hematopoiesis observed in this patient population^12^. In our study, hemoglobin levels were significantly lower in GD patients versus in healthy controls. This was in accordance with Rodic et al.^12^ findings, who reported anemia and thrombocytopenia to be the most frequently observed hematological findings in GD patients. This study also stated that leukopenia with predominant lymphocytopenia was a common feature, which may explain some of the immunological disturbances observed in GD patients^12^.

The disturbance in the secretion of cytokines regulating the differentiation and proliferation of lymphocytes may be an important contributing factor to the observed disturbance in the proportion of cytotoxic CD8 T-cells and NK cells^12^. Cytotoxic CD8 and NK cells are two different lymphocytes that have an important function in dealing with infections and in regulating the immune system^22^.

In our study, elevated proportions of CD8 cells were found in GD patients on regular ERT in comparison with among healthy controls. These results are comparable to those of Balreira et al.^23^, who examined GD patients both receiving ERT or not receiving it. Also, Limgala et al.^24^ and Zahran et al.^3^ reported similar results among GD patients receiving ERT. On the other hand, Lacerda et al.^9^ confirmed a reduction in the count of CD8+ T-cells among GD patients presenting with skeletal abnormalities. Also, Rodic et al.^12^ revealed that CD8+ T-cell counts were decreased in GD patients. Meanwhile, Sotiropoulos et al.^24^ observed an increase in CD8+ T-cells in GD patients, with no significant differences between those with and without skeletal abnormalities. Although this kind of observation contradicts what Lacerda et al.^9^ reported, it may be explained by the small number (n = 5) of GD patients with skeletal abnormalities that were included in that study^9^. A significant positive correlation was observed between the chitotriosidase enzyme level and CD8 cell count in GD patients. Despite the fact that the physiological activities of chitotriosidase are unknown, there is evidence that it is a component of innate immunity and may protect against diseases that include chitin, such as fungi, worms, and insects^25^. Chitotriosidase is a macrophage stimulation marker that is primarily produced by persistently activated tissue macrophages. Under healthy settings, leucocytes may also release plasma chitotriosidase. Patients with lysosomal storage diseases (as our cohort) have increased serum chitotriosidase activity^25,26^. Further research is needed to understand the correlations and role of chitotriosidase and CD8+ T-cells in GD.

The activated macrophages in patients with GD release cytokines that influence hematopoiesis; some of them may enhance the spread of B-cells with the production of antibodies, while others dampen the production of T-cells. Regular infusion of ERT is supposed to correct many of these cytokine disturbances in GD patients^12^. In the present study, the enrolled GD patients were receiving ERT, and their lymphocyte subsets may have been, to some extent, altered, marked by the improvement of the enzymatic defect and the reduction of splenomegaly. However, ERT in some research did not seem to correct or significantly affect immune cell dysregulation^24^. Autoantibodies against the protein part of the ERT were observed in 15% of patients with GD within the first year of its usage^27^, which might explain the variation in patients' response to ERT in different studies. Also, the time of starting the infusion of the ERT in each patient may impact their response^24^. To establish the validity of this hypothesis, more investigations and studies of lymphocyte subsets need to be performed before starting treatment with ERT and in larger groups of patients with consideration of the duration of ERT usage in GD patients.

Our study showed that the proportion of NK cells in GD patients is decreased in comparison with among healthy controls. The same results were obtained previously^11,28,29^. This decrease in NK cells may be due to continuous stimulation and chronic apoptosis and may be a risk factor for the B-cell malignancy and other lymphoid tumors previously reported in GD patients^1,29^. On the other hand, Limgala et al.^24^ observed no significant difference in the percentage of NK cells in GD patients under ERT versus healthy controls.

This study showed that the proportions of NK T-cells in both groups did not significantly differ (p = 0.272), which does not match with what Limgala et al.^24^ found, as these authors observed a significant increase in the proportion of CD3-expressing NK cells in GD patients relative to among controls (p = 0.0053). This might be explained by differences in the immune response to ERT of GD patients, with residual alteration in many patients' immune cells.

Limitations of this study could be the small number of included patients and healthy controls, as well as the conduct of a quantitative assessment of immune cells without accompanying functional assessment. Also, the included GD patients were all receiving ERT, and there were no blood samples available from the period prior to starting the ERT to ascertain the existence of residual alteration in immune cells after starting ERT. In addition, we were unable to investigate the levels of CD8 T-cells in a control group of children with no GD who have respiratory infections. It is, therefore, important to confirm these data in a larger patient and control cohorts exhibiting these features.

In conclusion, GD patients on regular ERT have increased CD8+ T-cell counts, predominantly Tc1, together with a reduction in the number of NK cells. These crucial immunological changes may contribute to some extent to the pathogenesis and the progression of GD.

## Abbreviations

GD: Gaucher disease
CD8: Cytotoxic T-cells
ERT: Enzyme replacement therapy
INF: Interferon
IL-1: Interleukin-1
NK: Natural killer cells
PBS: Phosphate-buffered saline

## References

[1] Zahran AM (2021). Dendritic cells and monocyte subsets in children with Gaucher disease. Pediatr. Res..

[2] Bettman N (2015). Impaired migration capacity in monocytes derived from patients with Gaucher disease. Blood Cells Mol. Dis..

[3] Zahran AM (2017). Activated and memory T lymphocytes in children with Gaucher disease. Arch. Immunol. Ther. Exp..

[4] Giraldo P (2016). Patients with type 1 Gaucher disease in Spain: A cross-sectional evaluation of health status. Blood Cells Mol. Dis..

[5] Barak V (1999). Cytokines in Gaucher's disease. Eur. Cytokine Netw..

[6] Pandey MK, Grabowski GA (2013). Immunological cells and functions in Gaucher disease. Crit. Rev. Oncog..

[7] Mittrücker HW (2014). Heterogeneity in the differentiation and function of CD8^+^ T cells. Arch. Immunol. Ther. Exp..

[8] Kaech SM, Cui W (2012). Transcriptional control of effector and memory CD8+ T cell differentiation. Nat. Rev. Immunol..

[9] Lacerda L (1999). T cell numbers relate to bone involvement in Gaucher disease. Blood Cells Mol. Dis..

[10] Bendelac A (2007). The biology of NKT cells. Annu. Rev. Immunol..

[11] Burstein Y (1987). Abnormalities of cellular immunity and natural killer cells in Gaucher's disease. J. Clin. Lab. Immunol..

[12] Rodic P (2014). Flow cytometric assessment of lymphocyte subsets in Gaucher type 1 patients. Blood Cells Mol. Dis..

[13] Salio M (2014). Biology of CD1- and MR1-restricted T cells. Annu. Rev. Immunol..

[14] Zigmond E (2008). NKT lymphocyte polarization determined by microenvironment signaling: a role for CD8+ lymphocytes and beta-glycosphingolipids. J. Autoimmun..

[15] Rhost S (2012). Immunomodulatory type II natural killer T lymphocytes in health and disease. Scand. J. Immunol..

[16] Hams E (2013). Cutting edge: IL-25 elicits innate lymphoid type 2 and type II NKT cells that regulate obesity in mice. J. Immunol..

[17] Matta MC (2018). Could enzyme replacement therapy promote immune tolerance in Gaucher disease type 1?. Blood Cells Mol. Dis..

[18] Shemesh E (2015). Enzyme replacement and substrate reduction therapy for Gaucher disease. Cochrane Database Syst. Rev..

[19] Brooks DA (2003). Significance of immune response to enzyme-replacement therapy for patients with a lysosomal storage disorder. Trends Mol. Med..

[20] Weinreb N (2008). A benchmark analysis of the achievement of therapeutic goals for type 1 Gaucher disease patients treated with imiglucerase. Am. J. Hematol..

[21] Regenboog M (2016). Hyperferritinemia and iron metabolism in Gaucher disease: Potential pathophysiological implications. Blood Rev..

[22] Nutt SL, Huntington ND (2019). Cytotoxic T lymphocytes and natural killer cells. Clinical Immunology.

[23] Balreira A (2005). Evidence for a link between sphingolipid metabolism and expression of CD1d and MHC-class II: Monocytes from Gaucher disease patients as a model. Br. J. Haematol..

[24] Limgala RP (2016). Time of initiating enzyme replacement therapy affects immune abnormalities and disease severity in patients with Gaucher disease. PLoS ONE.

[25] Wajner A (2004). Biochemical characterisation of chitotriosidase enzyme. Comparison between normal individuals and patients with Gaucher and Niemann-Pick diseases. Clin. Biochem..

[26] Van Eijk M (2005). Characterization of human phagocyte-derived chitotriosidase, a component of innate immunity. Int. Immunol..

[27] Kuhn A (2009). CD4(+) CD25 (+) regulatory T cells in human lupus erythematosus. Arch. Dermatol. Res..

[28] Braudeau C (2013). Altered innate function of plasmacytoid dendritic cells restored by enzyme replacement therapy in Gaucher disease. Blood Cells Mol. Dis..

[29] Schleinitz N (2010). Natural killer cells in human autoimmune diseases. Immunology.

